# Conserved targeting information in mammalian and fungal peroxisomal tail-anchored proteins

**DOI:** 10.1038/srep17420

**Published:** 2015-12-02

**Authors:** Judith Buentzel, Fabio Vilardi, Amelie Lotz-Havla, Jutta Gärtner, Sven Thoms

**Affiliations:** 1 University Medical Center, Department of Child and Adolescent Medicine, Robert Koch Str. 40, 37075 Göttingen, Germany; 2 Department of Molecular Biology, Humboldtallee 23, 37073 Göttingen, Germany; 3 Dr. von Haunersches Kinderspital, Lindwurmstr. 2a, 80337 München

## Abstract

The targeting signals and mechanisms of soluble peroxisomal proteins are well understood, whereas less is known about the signals and targeting routes of peroxisomal membrane proteins (PMP). Pex15 and PEX26, tail-anchored proteins in yeast and mammals, respectively, exert a similar cellular function in the recruitment of AAA peroxins at the peroxisomal membrane. But despite their common role, Pex15 and PEX26 are neither homologs nor they are known to follow similar targeting principles. Here we show that Pex15 targets to peroxisomes in mammalian cells, and PEX26 reaches peroxisomes when expressed in yeast cells. In both proteins C-terminal targeting information is sufficient for correct sorting to the peroxisomal membrane. In yeast, PEX26 follows the pathway that also ensures correct targeting of Pex15: PEX26 enters the endoplasmic reticulum (ER) in a GET-dependent and Pex19-independent manner. Like in yeast, PEX26 enters the ER in mammalian cells, however, independently of GET/TRC40. These data show that conserved targeting information is employed in yeast and higher eukaryotes during the biogenesis of peroxisomal tail-anchored proteins.

Peroxisome biogenesis requires the concerted action of a number of proteins termed PEX proteins or peroxins. These proteins form the import machinery for peroxisomal matrix proteins, and contribute to peroxisome membrane formation and to peroxisome inheritance^1^. The import of most peroxisome matrix proteins is dependent on PEX5, a soluble receptor that recognizes the peroxisomal targeting signal type 1 (PTS1). PMPs, on the other hand, can enter the peroxisomal membrane either via passage through the ER membrane, or post-translationally via a direct PEX19-dependent pathway. The peroxisome biogenesis factor PEX19 recognizes PMPs by their membrane PTS (mPTS) and, aided by PEX3, chaperones its cargo to and/or into the peroxisomal membrane. Cells are virtually devoid of peroxisomes when one of the peroxins PEX19, PEX3, or PEX16 is not functional^2^,^3^,^4^.

Cellular peroxisome formation is impaired in a number of genetic disorders, collectively termed peroxisome biogenesis disorders (PBD)^5^. These diseases are characterized by a deficiency of a peroxin leading to an inability to form mature, functional peroxisomes. *PEX1* is the most commonly affected gene in human PBD. PEX1 and PEX6 are ATPases of the AAA family^6^, members of which are often special chaperones or segregases, controlling the interaction of other proteins and/or membrane fusion processes^7^. Two different, but not necessarily exclusive functions have been described for AAA peroxins^8^. PEX6 and PEX1 are involved in recycling of PEX5 from the peroxisomal lumen into the cytosol^9^ and biogenesis of peroxisomes from precursor membrane structures by fusion of immature peroxisome precursors^10^,^11^. Import of peroxisomal matrix proteins requires a translocon that cycles PEX5 and its cargo into the peroxisome. Two components form this import machinery: the docking and the RING complex. In yeast, these complexes are stored separately in two distinct pre-peroxisomal vesicles. Upon vesicle fusion during peroxisome biogenesis both RING and docking complex form the peroxisomal translocon, thus enabling peroxisome matrix protein import^11^,^12^.

In yeast, the PMP Pex15 anchors Pex1 and Pex6 to the membrane^13^. In mammals PEX26 is the membrane anchor for PEX1 and PEX6^14^. Both, Pex15 and PEX26, are tail-anchored (TA) proteins, integral membrane proteins with a single transmembrane domain (TMD) located at the C-terminus^14^,^15^. The TMD of TA proteins necessitates post-translational import into its target membrane^16^. TA proteins destined for the ER can enter this organelle by several pathways. The signal recognition particle (SRP) is able to recognize some TA proteins after translation^17^. Short secretory proteins use the Sec62/63 channel for translocation into the ER^18^. The chaperones Hsp40 and Hsc70 do also stabilize TA proteins post-translationally and mediate ER targeting^19^. But the majority of TA proteins is targeted to the ER via the GET/TRC40-pathway^20^. In yeast Get3 recognizes, binds, and targets the TA protein to the ER^21^. Upon interaction with the Get1/Get2-receptor complex Get3 releases its cargo, which inserts into the ER membrane^22^,^23^,^24^,^25^,^26^. TRC40 is the mammalian homologue of Get3 27. Insertion of TA proteins into the ER is facilitated by the interaction of TRC40 with a membrane receptor complex formed by WRB^24^,^28^ and CAML^29^,^30^.

PEX26 and Pex15 pose an interesting puzzle: while both are tail-anchored and share the same function, they share no sequence similarity. Pex15 is either a very distant homologue of PEX26 that cannot be recognized due to extreme sequence divergence (divergent evolution), or it has evolved independently with a similar function and membrane topology (convergent evolution). Whereas it was shown that Pex15 enters the ER dependent on the GET-pathway before being targeted to the peroxisome^15^,^22^, PEX26 is reported to target PEX19-dependently to the peroxisome directly after its synthesis in the cytosol without involvement of the ER^31^,^32^. As Pex15 and PEX26 share a common function, we wanted to analyze if both proteins have common, conserved targeting features. Our results show that PEX26, like Pex15, is integrated into the ER. Furthermore Pex15 and PEX26 are targeted to peroxisomes if expressed in human cells and *S. cerevisiae* respectively, arguing for evolutionarily conserved membrane targeting information in these proteins.

## Results

### Conserved peroxisome targeting information in the membrane protein PEX26

**Figure 1:**
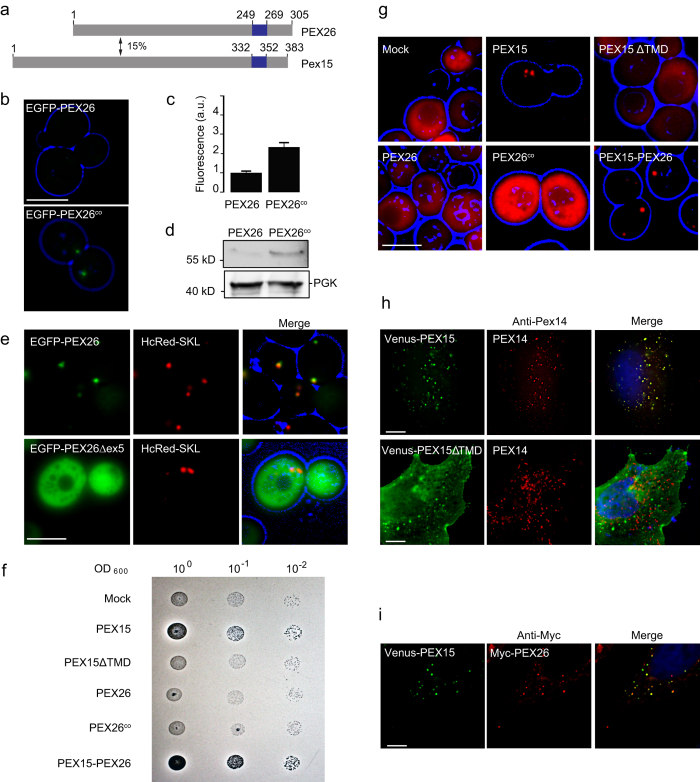
Peroxisome targeting information in PEX26 and Pex15 is evolutionarily conserved. (**a**) Human PEX26 and yeast Pex15 are functionally similar tail-anchored proteins with only 15% amino acid identity (pairwise alignment Emboss Needle default settings). Predicted TMDs (Topcons^62^) are marked in blue. TMDs are followed by the luminal amino acids, the so-called C-segment. (**b**–**d**) Improved expression of PEX26 in yeast after codon-optimization. GPD promoter-driven expression of PEX26 and codon-optimized PEX26^co^ in wild-type yeast cells. PEX26 was labelled at the N-terminus with the green fluorescent protein EGFP. (**b**) Direct fluorescence of EGFP-PEX26 and EGFP-PEX26^co^. Cell contours are shown in blue (widefield images). Bar = 10 μm. (**c**) Quantification of (**b**). The expression of PEX26^co^ increased by factor 2.3. N = 94 and 102. Error bar = s.e.m. P < 0.0001 (unpaired t-test). (**d**) Western blot of logarithmically grown cells with an anti-GFP antibody. Full-size blots are presented in Supplementary Figure S1. (**e**) PEX26 localizes to peroxisomes in yeast. Co-localization with the peroxisome marker HcRed-SKL. PEX26Δex5 lacks the TMD encoded by exon 5 and shows cytosolic localization. Bar = 5 μm. (**f**) The TMD and C-segment of PEX26 can functionally replace the TMD and C-segment of Pex15 in yeast. PEX26 cannot restore peroxisome maturation in Δ*pex15* yeast. Growth assay on oleate plates of Δ*pex15* cells transformed with plasmids expressing Pex15, Pex15ΔTMD, PEX26, PEX26^co^ or Pex15-PEX26. Halos indicate oleate consumption. (**g**) The Pex15-PEX26-fusion protein is able to restore peroxisomal matrix protein import of HcRed-SKL in Δ*pex15* yeast cells. Pex26 and PEX26^co^ fail to re-establish peroxisomal matrix protein import. Co-expression of Pex15, Pex15ΔTMD, PEX26, PEX26^co^ or Pex15-PEX26 with HcRed-SKL. Bar = 5 μm. (**h**) Yeast Pex15 is imported into peroxisomes in HeLa cells. The peroxisomal targeting information of Pex15 is situated in the TMD and the C-segment. Pex15ΔTMD remains cytosolic. Immunofluorescence of Venus-Pex15 and Venus-Pex15ΔTMD with antibodies directed against the peroxisomal membrane protein PEX14. Bar = 10 μm. (**i**) PEX26 and Pex15 co-localize in the same population of peroxisomes in HeLa cells upon co-expression. Co-transfection with Venus-Pex15 and Myc-PEX26 and immunofluorescence with anti-Myc antibody directed against Myc-PEX26. Bar = 10 μm.

Mammalian PEX26 and yeast Pex15 share a similar function in AAA peroxin binding and membrane recruitment, and both are TA proteins^14^. Yet both proteins show only 15% amino acid identity, indicating that they might not be homologous (Fig. 1a). In the light of their similar function but their dissimilar primary structure we wanted to investigate if both proteins also share common features in their targeting itineraries and mechanisms, and if they follow general mechanisms of membrane targeting. We therefore expressed human PEX26 in yeast fused with an N-terminal enhanced green fluorescent protein (EGFP)-tag under the control of the glyceraldehyde-3-phosphate dehydrogenase gene (GPD) promoter. Although the GPD promoter usually leads to strong overexpression, PEX26 showed very low expression in wild-type *S. cerevisiae* (Fig. 1b). We speculated that unfavorable codon usage of the human protein is the cause for the low expression in yeast. Therefore we mutated the seven uncommon CGG arginine codons to CGC (nucleotide positions 93, 156, 180, 194) or AGG (nucleotide positions 496, 571, 871), which are more common codons for arginine in yeast^33^. The resulting codon-optimized construct *PEX26*^*co*^ expressed at twofold intensity in yeast compared to the original *PEX26* construct (Fig. 1b,c). Improved PEX26 expression from the codon-optimized construct was confirmed by Western blot (Fig. 1d).

Next we studied the intracellular localization of human PEX26 in yeast. When we expressed PEX26 fused to the C-terminus of the EGFP, the protein accumulated in puncta indicative of peroxisomal localization (Fig. 1b,e). Co-expression of EGFP-PEX26 with the peroxisome matrix marker HcRed-SKL led to co-localization of both fluorescent fusion proteins (Fig. 1e). We therefore conclude that human PEX26 is targeted to peroxisomes in *S. cerevisiae*. The natural variant PEX26Δex5 lacks the TMD which is encoded by exon 5 34. Expression of PEX26Δex5 fused with an N-terminal EGFP leads to cytosolic localization (Fig. 1e) indicating that the TMD is needed for peroxisomal targeting. To test this further, we cloned and expressed the TMD of PEX26 together with the C-segment as a fusion protein with N-terminal EGFP. The fusion protein localized to the peroxisome, although it is expressed at a low level (Supplementary Figure S3).

Given the surprising localization of PEX26 in yeast peroxisomes, we investigated if PEX26 could complement *pex15*-deficiency in yeast. Yeasts with defective Pex15 lack mature peroxisomes^15^ and are thus not able to grow on oleate medium^35^. Expression of Pex15 allowed growth on oleate, but expression of *PEX15*ΔTMD and PEX26 could not complement the Δ*pex15* strain (Fig. 1f). To test if the C-terminal TMD and peroxisomal targeting information of PEX26 combined with the cytosolic domain of Pex15 would restore peroxisomal function, we designed a Pex15-PEX26 fusion protein comprising the cytosolic N-terminal part of Pex15 (amino acids 1 to 331) and the TMD with the C-terminus of PEX26 (amino acids 252 to 305). This fusion protein was expressed in the Δ*pex15* strain and allowed growth on oleate (Fig. 1f). Then we co-expressed the constructs used for the oleate assay with HcRed-SKL. Peroxisomal localization of HcRed-SKL and thus functional peroxisomes were only observed in the Δ*pex15* strains expressing Pex15 or the Pex15-PEX26 fusion protein (Fig. 1g). These experiments show that the cytosolic domain of PEX26 cannot replace the cytosolic domain of Pex15, but C-termini of PEX26 and Pex15 share common targeting information that allow peroxisomal targeting and restoration of peroxisome maturation in the Δ*pex15* strain. The peroxisomal localization of EGFP-PEX26 in *S. cerevisiae* suggests a conserved targeting signal for this peroxisomal protein between mammalian cells and yeast. In all subsequent experiments with PEX26 in yeast, we used this codon-optimized form, and we will refer to *PEX26*^*co*^ as *PEX26*.

To further investigate this conserved targeting process, we conversely expressed Venus-Pex15 in HeLa cells and analyzed its localization by direct fluorescence and immunofluorescence. The majority of Venus-Pex15 localized in puncta that co-labelled with the peroxisomal marker PEX14 (Fig. 1h). These results show that, like PEX26 in *S. cerevisiae*, Pex15 localizes to peroxisomes in HeLa cells.

We then asked if Pex15 and PEX26 localized into the same or different populations of human peroxisomes. Myc-PEX26 and Venus-Pex15 were co-expressed in HeLa cells and analyzed by combined immunofluorescence and direct fluorescence. Both, PEX26 and Pex15 localized into puncta that coincided with each other showing that Pex15 and PEX26 are targeted into the same population of peroxisomes (Fig. 1i).

PMPs are thought to share a — yet poorly defined — targeting signal found near the TMD, called membrane PTS (mPTS)^36^. We therefore think that the conserved targeting information, that allowed the peroxisomal targeting of Pex15 in HeLa cells and PEX26 in yeast, is localized near the C-terminus, comprising the TMD and the luminal segment. This is supported by the complementation of the fusion protein Pex15-PEX26 in Δ*pex15* yeast. When we expressed Pex15 without the TMD and the luminal segment (Venus-Pex15ΔTMD) in HeLa-cells, the protein localized to the cytosol and could not enter the peroxisome which further supports this hypothesis (Fig. 1h). Gray-scale representations of single channel images of Fig. 1h,i are in Supplementary Figure S2.

Like PEX26 in S. cerevisiae, yeast Pex15 localizes to peroxisomes in HeLa cells. The protein is targeted to the same sub-population of peroxisomes like human PEX26. As HeLa cells comprise functional peroxisomes that require PEX26, this observation indicates that Pex15 was inserted into mature peroxisomes. Pex15 and PEX26 share a targeting signal, which is conserved amongst species.

### The C-terminus of PEX26 gets exposed to the endoplasmic reticulum lumen

**Figure 2:**
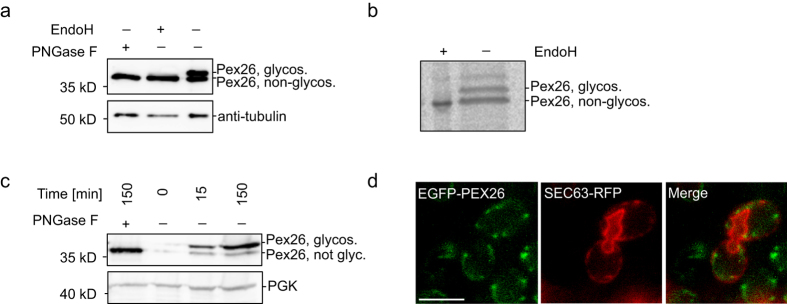
The C-terminus of PEX26 becomes exposed to the endoplasmic reticulum. (**a**) PEX26-ops enters the ER in HeLa cells. Glycosylation is used as marker for ER integration. Western blot. Samples were taken 48 hours after transfection with PEX26-ops. The slower migrating band indicates glycosylation. Controls: lysates were treated with the deglycosylating enzymes PNGase F and EndoH. (**b**) ER entry of PEX26-ops within 30 min. Autoradiograph. HeLa cells expressing PEX26-ops were labelled for 30 min with [^35^S]-methionine and [^35^S]-cysteine. Cells were lysed and PEX26-ops was immunoprecipitated with anti-opsin antibody. (**c**) PEX26-ops glycosylation indicates that the luminal domain is exposed to the ER in yeast. GAL1 promoter-driven expression of PEX26-ops in yeast is induced for 15 or 150 min. After 150 min, the majority of the PEX26-ops is glycosylated. (**d**) EGFP-PEX26 localizes to the ER in the absence of peroxisomes. Expression of EGFP-PEX26 in Δ*pex19* yeast. Close association of EGFP-PEX26 with the ER marker Sec63-RFP. Bar = 5 μm. Full-size blots and autoradiograph are presented in Supplementary Figure S1.

Pex15 traffics to the peroxisome via the ER in a GET-dependent manner^22^, whereas the targeting of PEX26 was shown to be independent of Get3-homologue TRC40 31. Given the similar peroxisome-targeting properties of Pex15 and PEX26, we wanted to study the earlier steps of PEX26 targeting in mammalian and in yeast cells and asked if PEX26 enters the ER. PEX26 was tagged with a short opsin-tag that can be glycosylated in the ER lumen to monitor ER-passage^37^. Samples were taken 48 hours after transfection of HeLa cells with PEX26-ops. Controls were treated with the deglycosylating enzymes EndoH and PNGase F. The control samples showed a single band on Western blot, whereas in untreated samples two bands were detected (Fig. 2a). The lower band migrated at the same level as the deglycosylated sample, thus representing the non-glycosylated protein. The upper band showed a lower mobility due to glycosylation. We conclude that PEX26 entered the ER.

To confirm these results we designed a radio-pulse label experiment. HeLa cells were transfected with PEX26-ops. Twenty-four hours after transfection, cells were labelled with [^35^S]-methionine and [^35^S]-cysteine for only 30 min and PEX26-ops was immunoprecipitated with an anti-opsin antibody. PEX26-ops appeared as a double band on the autoradiograph (Fig. 2b). A control with EndoH showed that the lower band corresponded to the deglycosylated form, whereas the upper band in the gel is the glycosylated protein (Fig. 2b). The short pulse was thus sufficient to monitor PEX26 glycosylation in the ER.

To investigate PEX26 trafficking in yeast, the C-terminus of PEX26 was tagged with a short opsin-tag codon-optimized for expression in yeast that would allow glycosylation if PEX26 passed the ER in *S. cerevisiae*. PEX26-ops was expressed under the control of the galactokinase (GAL1) promoter in wild-type cells for up to 150 min. A control was treated with PNGase. PEX26 migrated as double band, the lower coincided with the deglycosylated form, and the upper was only detected in the untreated sample (Fig. 2c). The mobility shift must thus be due to glycosylation, indicating that PEX26 targets to the ER in yeast also in the pulse-experiment.

Next we co-expressed GAL1-driven EGFP-PEX26 with the ER-marker Sec63-RFP in Δ*pex19* cells to assess the localization of PEX26 in the absence of peroxisomes in a pulse-chase experiment. One hour after the pulse, EGFP-PEX26 was found in close association with the ER-marker (Fig. 2d) indicating that PEX26 accumulates in an ER-proximate compartment. These results also suggest that Pex19 is not required for targeting PEX26 to the ER. Similar to Pex15, PEX26 is inserted into the ER in *S. cerevisiae*^15^,^38^. Furthermore we could show that the luminal part of the protein gets exposed to the ER lumen in HeLa cells.

### Human PBD fibroblasts with mutations in PEX26

**Figure 3:**
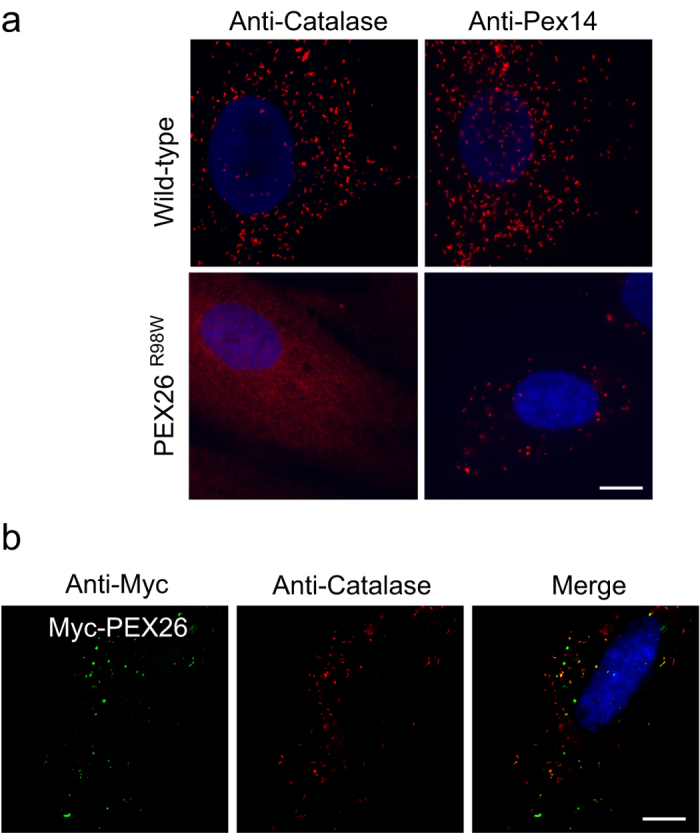
Human PBD fibroblasts with a mutation in *PEX26*. (**a**) *PEX26* deficiency leads to impaired peroxisome maturation. Fibroblasts from a control and from a patient with PEX26^R98W^ mutation were probed with antibodies directed against the peroxisomal matrix protein catalase and the peroxisomal membrane protein PEX14. No catalase import in patient fibroblasts. Apparent peroxisome size is increased in the patient fibroblasts, but peroxisomes are less abundant. Bar = 10 μm. (**b**) Expression of Myc-PEX26 in PEX26^R98W^ fibroblasts partially restores peroxisome maturation and import of catalase. Catalase is largely localized to the peroxisome, but peroxisome number is still reduced. Bar = 10 μm. Gray-scale representations of single channel images of this figure are in Supplementary Figure S2.

PEX26 contributes to peroxisome biogenesis by anchoring PEX6 and PEX1 to the membrane of the peroxisome. The development of a mature import-competent peroxisome is not possible in the absence of PEX26 3,9. To assess the cellular phenotype of *PEX26* deficiency we stained skin fibroblasts from a patient with a homozygous *PEX26* C292 > T (R98W) mutation for the peroxisomal matrix protein catalase. As expected, the protein showed cytosolic localization (Fig. 3a). When we stained for the PMP PEX14 round plump puncta were observed. They were larger in size and smaller in number than peroxisomes found in the control and clustered mainly in the cell center and close to the nucleus. These immature peroxisomes were not able to import catalase (Fig. 3a). In control fibroblasts, catalase was localized to defined puncta that were more evenly distributed within the cell (Fig. 3a). We tested if our *PEX26* construct was able to restore peroxisome maturation upon introduction in *PEX26*^*−/−*^-deficient fibroblasts. We transfected *PEX26*-deficient patient fibroblast cells with Myc-PEX26. Catalase was used as a marker for peroxisome biogenesis. In the transfected cell, catalase is largely localized to the peroxisome, but peroxisome number remains reduced. The construct thus partially restored catalase import in the deficient cell line (Fig. 3b).

### PEX26 targeting to the ER is dependent on the GET machinery

Next we wanted to analyze if the GET-pathway which is responsible for inserting Pex15 into the ER^39^, is also used by PEX26. As TA proteins employ a broad range of mechanisms that lead to their targeting to the ER^24^,^40^ we also tested for a possible dependency on post-translational targeting factors.

**Figure 4:**
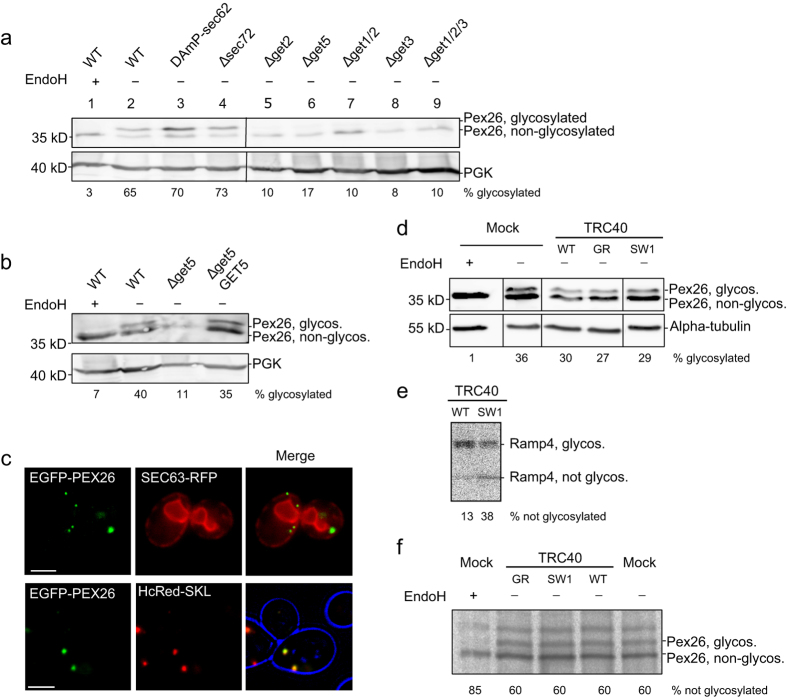
PEX26 targeting to the ER is dependent on the GET machinery. (**a**–**c**) GAL1 promoter-driven pulse expression of PEX26-ops in yeast. Opsin glycosylation reports entry into the ER. (**a**) GET-dependent but SEC62/SEC72-independent entry of PEX26-ops into the ER in yeast. Strains defective in post-translational ER targeting (DAmP-*sec62*, Δ*sec72*)^63^ or the GET pathway were transformed with PEX26-ops and expression was induced for 120 min. Western blot with anti-opsin antibodies. Both halves of each panel were cropped from the same blot. Quantifications of band intensities can be found in Supplementary Table S1 (n ≥ 3). (**b**) Extrachromosomal GET5-expression restores ER entry of PEX26-ops in Δ*get5*-yeasts. Induction for 120 min. Western blot with anti-opsin antibodies. (**c**) EGFP-PEX26 does not integrate into the ER if the GET-pathway is impaired, but co-localizes with the peroxisomal marker HcRed-SKL. Co-expression of EGFP-PEX26 with Sec63-RFP ER marker or peroxisomal marker HcRed-SKL in a Δ*get3* strain. Bar = 5 μm. (**d**–**f**) Integration of PEX26-ops into the ER is TRC40-independent. Co-transfection of PEX26-ops with TRC40^WT^, TRC40^SW1^, TRC40^GR^ or mock plasmid. (**d**) TRC40 and TRC40 mutants do not influence ER targeting of PEX26-ops. Western blot. HeLa cells were co-transfected for 15 hours with PEX26-ops and mock, TRC40 or TRC40 mutant. All parts of each panel were cropped from the same blot. (**e**,**f**) TRC40-independent ER targeting of PEX26. Overexpression of the dominant TRC40 mutant SW1 impairs ER entry of Ramp4-ops but not of PEX26. HeLa cells co-expressing (**e**) Ramp4-ops or (**f**) PEX26 and TRC40 variants were labelled for 15 min with [^35^S]-methionine and [^35^S]-cysteine. (**e**) Ramp4-ops and (**f**) PEX26 were immunoprecipitated with anti-opsin antibodies and analyzed by autoradiography. Band intensities were quantified. Full-size blots and autoradiograph are presented in Supplementary Figure S1.

PEX26-ops was expressed in different *get*-deficient strains, and in DAmP-*sec62* and Δ*sec72* yeasts. Protein expression under the control of the GAL1 promoter was induced by shifting de-repressed cells to galactose. PEX26-ops, when expressed in the wild-type control strain, appeared as a double band in the Western blot (Fig. 4a, lane 2). Double bands representing both glycosylated and non-glycosylated protein were also present in *sec62*- and *sec72*-deficient strains. There was no change of glycosylation levels observed between the PEX26-ops expressed in DAmP-*sec62* or Δ*sec72* and the control (Fig. 4a, lanes 3 and 4, see also Supplementary Table S1). In all *get*-deficient strains, however, the majority of PEX26 migrated in the fraction of the lower band corresponding to the non-glycosylated PEX26 (Fig. 4a). Less than 20% of PEX26 became glycosylated in all strains lacking one or several components of the GET-pathway acting at different stages of TA protein biogenesis (Fig. 4a, lanes 5 to 9). When we transformed the *get5*-deficient strain with a *GET5*-expressing plasmid, the pathway was rescued and PEX26 glycosylation was restored (Fig. 4b). We conclude that ER-targeting of PEX26 is dependent on the GET-pathway. When EGFP-PEX26 was pulse-expressed in a Δ*get3* strain, the protein localized into green puncta that did not coincide with the ER, but with the peroxisomal marker HcRed-SKL indicating that PEX26 can reach peroxisomes in the absence of GET3 (Fig. 4c).

Taken together, the data indicate a GET-dependent but non-essential ER targeting of PEX26. PEX26 can eventually reach peroxisomes even if the GET-pathway is impaired. ER integration of PEX26 also appears to be independent of the post-translational Sec62 and Sec72 translocon subunits.

In the light of the GET-dependency of PEX26 in *S. cerevisiae* we wanted to investigate if ER integration in HeLa cells was dependent on the mammalian Get3 homologue TRC40. HeLa cells were co-transfected with PEX26-ops and TRC40 or dominant negative mutants of TRC40 previously reported to impair TA protein targeting in HeLa cells^39^. PEX26-ops was detected by Western blot. Glycosylation was not altered between the control and samples overexpressing TRC40 or TRC40 mutants (Fig. 4d). To be able to identify smaller changes in PEX26 glycosylation, we performed a radio-pulse experiment co-expressing the dominant TRC40 mutant SW1 that impedes ER entry of the TA protein Ramp4-ops (Fig. 4e). PEX26-ops was precipitated through the opsin-tag from radio-labelled cells co-transfected with PEX26-ops and TRC40. Similar to Western blot analysis, the autoradiograph indicated that PEX26 glycosylation is not influenced by co-expression of TRC40 or dominant TRC40 mutants (Fig. 4f). These findings confirm previous microscopy studies showing that the ER targeting of PEX26 in mammalian cells is independent of the TRC40-pathway^31^.

### In yeast PEX26 glycosylation occurs before Pex19-dependent peroxisome formation

**Figure 5:**
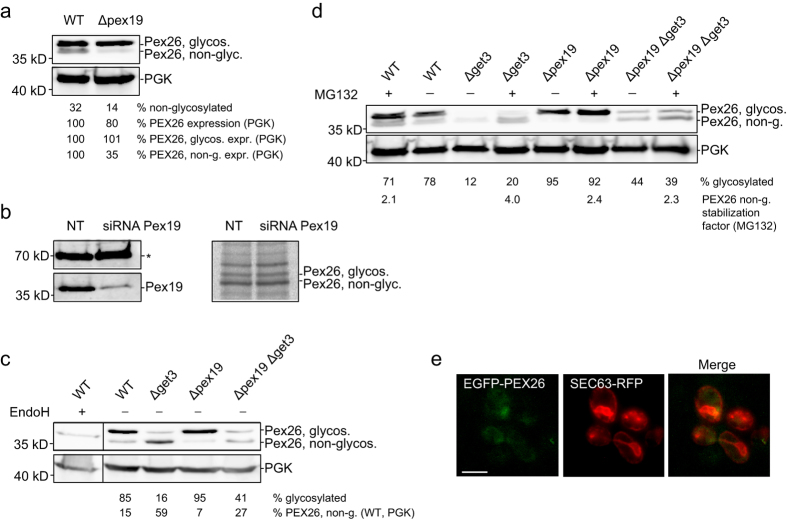
The Pex19-dependent steps occur after or independently of ER entry of PEX26. (**a**,**b**) ER integration of PEX26-ops is independent of PEX19, both, in HeLa and in yeast cells. (**a**) Knock-out of *PEX19* does not affect the ER entry of PEX26-ops but affects the level of non-glycosylated PEX26. Expression of PEX26-ops in Δ*pex19* cells for 120 min. To compare protein expression in wild-type and Δ*pex19* PEX26-ops expression is stated in relation to PGK levels. ‘%PEX26 expression’ is the total amount of PEX26 relative to PGK. ‘%PEX26, glycos. expr.’ and ‘%PEX26 non-g. expr.’ are the levels of glycosylated and non-glycosylated forms relative to PGK. WT was set to 100%. Quantifications show that only the non-glycosylated PEX26-ops is reduced to 35% in Δ*pex19* cells. (**b**) PEX19 does not influence ER entry of PEX26-ops in mammalian cells. HeLa cells expressing PEX26-ops were treated for 60 hours with siRNA directed against PEX19 or with non-targeting siRNA. For radiolabelling, cells were grown for 15 min with [^35^S]-cysteine and [^35^S]-methionine before immunoprecipitation of PEX26-ops. Western blot (left): Knockdown of PEX19. The asterisk marks a nonspecific band as a loading control. Autoradiograph of radio-pulse experiment (right): Pex26-ops glycosylation is not reduced. (**c**) Pex19 stabilizes cytosolic PEX26-ops in the absence of Get3. The additional Δ*pex19* knockout in Δ*get3* reduces the non-glycosylated form of PEX26-ops from 59% to 27%. ‘%PEX26 non-g. (WT, PGK)’ is relative to wild-type, normalized by PGK protein expression. Both parts of each panel were cropped from the same blot. (**d**) Pex19 and Get3 stabilize cytosolic PEX26-ops. Non-stabilized PEX26-ops is degraded by the proteasome. Cells treated with 30 μM MG132 show a 2.1- to 4-fold increase of non-glycosylated, cytosolic PEX26-ops in wild-type, Δ*pex19*, Δ*get3*, and Δ*pex19*Δ*get3* strains. (**e**) Pex19 and Get3 are required for organelle targeting of PEX26. Localization of EGFP-PEX26 in Δ*pex19*Δ*get3* yeast. GAL1 promoter-driven expression was recorded 80 min after chase. EGFP-PEX26 does not co-localize with the ER marker Sec63-RFP. Bar = 5 μm. Full-size blots and autoradiograph are presented in Supplementary Figure S1.

PEX19 plays a crucial role in targeting of PMPs to the peroxisome^36^,^41^ and has been shown to support the trafficking of PEX26 to peroxisomes^31^,^42^. We now find that PEX26 enters the ER. Therefore we wanted to investigate if PEX19 is involved in the integration of PEX26 into the ER or at later steps of peroxisome formation. We expressed PEX26 in *pex19*-deficient yeast cells. Again, the protein was controlled by the GAL1 promoter and induced in a pulse experiment. In the wild-type, 32% of the PEX26 remained without glycosylation. In Δ*pex19*, only 14% were not glycosylated (Fig. 5a). The expression of PEX26 dropped by 20% relative to the control protein PGK. This reduction in the total amount of PEX26 can be attributed to the reduction of the not-glycosylated PEX26, because the amount of glycosylated PEX26 remains unchanged (Fig. 5a). This observation is in agreement with a role of Pex19 stabilizing PMPs in the cytosol^2^.

PEX26 was then expressed in HeLa cells treated with siRNA targeting PEX19. The knock-down reduced PEX19 protein by 81% (Fig. 5b). Cells were analyzed in a radio-pulse experiment with [^35^S]-methionine and [^35^S]-cysteine. PEX26-ops glycosylation level was not altered between cells treated with siRNA or without (Fig. 5b). These data indicate the ER entry of PEX26 is independent of PEX19.

Our data in yeast show that PEX26 targeting to the ER is dependent on Get3, but not on Pex19. However, Get3 and Pex19 are chaperones affecting the targeting of PEX26. Therefore we wanted to investigate the functional relationship of these proteins in PEX26 targeting. We expressed PEX26 in Δ*get3* and in Δ*pex19* strains and compared the glycosylation pattern in these mutants with a Δ*pex19*Δ*get3* knock-out. Expression in Δ*get3* confirmed that the majority of PEX26 remained non-glycosylated, whereas glycosylation was observed again when expressing PEX26 in Δ*pex19*-cells (Fig. 5c). The double knock-out showed a reduction of total PEX26 compared to the wild-type and the single knock-out strains (Fig. 5c). The level of glycosylated PEX26 in the double knock-out was similar to Δ*get3* (Fig. 5c). These results suggest that the absence of *PEX19* does not interfere with the Get3-dependent targeting of PEX26, but that Pex19 is able to stabilize the cytosolic PEX26 that accumulates in the absence of Get3.

In order to investigate if the reduction of total PEX26 in Δ*pex19*Δ*get3* cells was due to protein degradation we repeated the pulse-experiment and added 30 μM of the proteasome inhibitor MG132 to yeast cultures during induction of PEX26-ops expression. We observed an increase of PEX26-ops, both glycosylated and non-glycosylated, in all samples treated with the proteasome inhibitor. For example, the non-glycosylated form in Δ*pex19* cells increased by a factor of 2.4 (Fig. 5d). We conclude that both, Pex19 and Get3, stabilize PEX26-opsin after translation in yeast.

We expressed GAL1-driven EGFP-PEX26 in Δ*pex19*Δ*get3* to visualize the localization of PEX26. 80 min after the pulse the majority of EGFP-PEX26 localized in the cytosol (Fig. 5e). ER-localization or a similar behavior like in Δ*get3*-cells (EGFP-PEX26 in puncta, like in Fig. 4c) was not observed. We conclude that Pex19 and Get3 are both responsible for targeting PEX26 in yeast.

**Figure 6:**
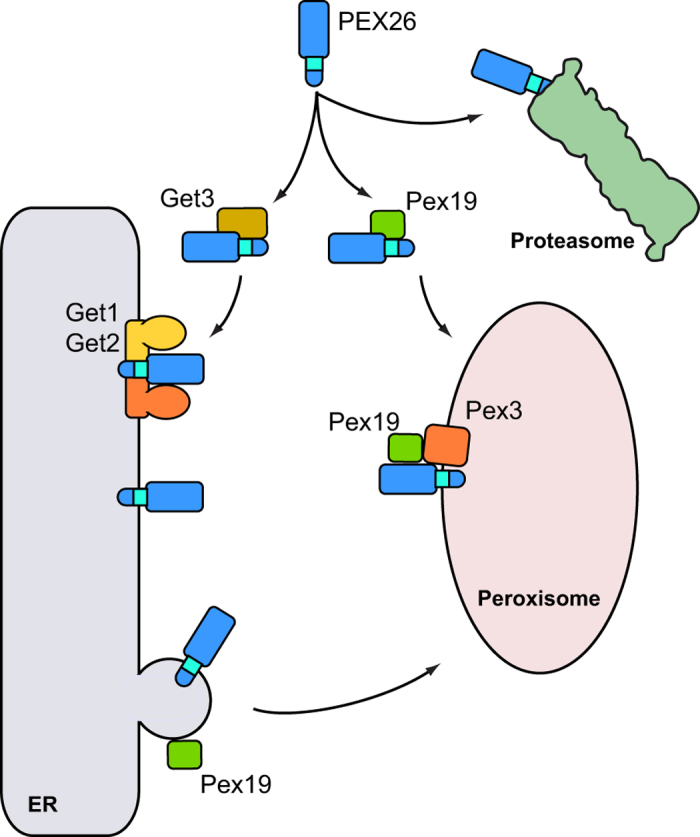
Model of PEX26 targeting. TA proteins are translated in the cytosol and require chaperone systems for post-translational targeting. The peroxisomal TA protein PEX26 is recognized by Get3 in the cytosol and directed to the ER. The receptor complex consisting of Get1 and Get2 mediates the insertion of PEX26 into the ER membrane. In the membrane, PEX26 is sorted into pre-peroxisomal vesicles that require Pex19 for budding from the ER. In the absence of *GET3,* the majority of cytosolic PEX26 is stabilized by the PMP chaperone Pex19 and directly targeted to the peroxisome, where an interaction of Pex19 and Pex3 mediates the integration of PEX26 into the peroxisomal membrane. If both, Get3 and Pex19, are inactivated, PEX26 is degraded by the proteasome.

Taken together, in yeast PEX26 is targeted GET-dependently to the ER. When the GET-pathway is blocked, the cytosolic pool of PEX26 is further stabilized by the yeast Pex19. The recognition of PEX26 by yeast Pex19 is another conserved feature of the peroxisome biogenesis machinery. When Pex19 is deficient in addition to Δ*get3*, the expression and stability of PEX26 is further decreased. These data therefore suggest that the ER-targeting of PEX26 occurs before Pex19-dependent peroxisome formation (Fig. 6).

## Discussion

### The challenge of tail-anchored protein targeting to the peroxisome

Our understanding of TA protein targeting to the secretory pathway or the outer mitochondrial membrane has advanced considerably in recent years^24^,^25^,^40^,^43^. However, how TA proteins are integrated into the peroxisomal membrane is poorly understood. The TA proteins yeast Pex15 and mammalian PEX26 are PMPs involved in the recruitment of the AAA-type ATPases PEX1 and PEX6 to the maturing peroxisome^13^,^14^. Pex15 and PEX26 are thus functionally very similar despite showing only a low similarity in their amino acid sequence. The peroxisome targeting of both proteins is dependent on PEX19^32^,^44^. However, Pex15 is also known for its ability to enter the ER as a substrate of the GET-pathway^15^,^22^, whereas the targeting PEX26 is independent of the mammalian Get3 homologue TRC40 31.

In this study we wanted to revisit the targeting of these two proteins. We analyzed PEX26 in mammalian cells, and we codon-optimized PEX26 for expression in yeast. Cross-expression of PEX26 in yeast and Pex15 in HeLa cells showed that both exogenously expressed proteins are targeted to peroxisomes. This suggests that Pex15 and PEX26 could be targeted by a common, conserved membrane targeting signal.

### Targeting of the peroxisomal membrane protein PEX26 to the ER

PEX19 is thought to recognize PMPs shortly after their translation in the cytosol and to subsequently target them to the peroxisomal membrane^2^,^45^,^46^. However this model does not consider that various PMPs including Pex15 are targeted to the ER before entering the peroxisomal membrane^47^,^48^. To study if PEX26 enters the ER we tagged the protein with an opsin-tag that becomes glycosylated once the C-terminal domain faces the lumen of the ER. Surprisingly, PEX26 was glycosylated both in *S. cerevisiae* and HeLa cells, mirroring the behavior of Pex15. We conclude that passage through the ER is another common feature shared by Pex15 and PEX26. Generally, ER-targeting of TA proteins is dependent on the GET/TRC40-pathway, on a post-translational SRP-dependent pathway, or on chaperones of the Hsc70/Hsp40 family^40^. We were able to show that PEX26 can enter the ER in *S. cerevisiae* independently of the SEC-translocon subunits Sec62 and Sec72. This seems reasonable as the ER translocon containing Sec62 and Sec63 is specialized on post-translational transport of short secretory proteins comprising less than 80 amino acids^18^. In *S. cerevisiae* however PEX26 uses the GET-pathway for targeting to the ER similar to its yeast analog Pex15 22. Nevertheless, PEX26 can eventually reach peroxisomes even in absence of a functional GET-pathway in agreement with the postulated redundancy of TA protein biogenetic pathways^43^ and/or by spontaneous insertion^16^. Accordingly, in mammalian cells, dedicated but yet unidentified chaperones could shuttle peroxisomal TA proteins to the ER membrane in a TRC40-independent manner as shown by our experiments that confirmed TRC40-independent targeting of PEX26 in mammalian cells^31^.

Contrary to other models of PEX26 targeting^31^, the finding that PEX26 can enter the ER independent of the TRC40 pathway shows that GET/TRC40-independence cannot be used as argument against the involvement of the ER in peroxisome biogenesis routes. One possible reason for TRC40-independence of PEX26 might be that the TMD hydrophobicity of PEX26 is too low for recognition by TRC40, similarly to PEX26 from *Neurospora crassa*^49^. Human PEX26 has a more hydrophobic TMD than Pex15 49, which is a regular substrate of the GET-pathway. The higher hydrophobicity of human PEX26 might therefore be the reason why transport and insertion of PEX26 into the ER is GET-dependent in yeast. The effect of PEX16, a PMP found in mammalian but not in yeast cells, might also contribute to the differences in ER targeting of PEX26 in yeast and human. Overexpressed PEX16 was recently reported to recruit the TMD and the C-segment of PEX26 to the ER^50^. Although the signal recognized by PEX16 is not known, one could speculate that PEX16 is involved in the targeting of PEX26 in a manner similar to Get3 in yeast.

### The role of PEX19 in PMP targeting to the ER and the peroxisome

We tested the hypothesis that PEX19 is the chaperone that would mediate PEX26 entry into the ER. PEX26 was expressed in a Δ*pex19* yeast strain and in a [^35^S]-pulse experiment in HeLa cells treated with siRNA directed against *PEX19*. In both cases the levels of PEX26 glycosylation were not altered compared to wild-type. We conclude that PEX19 does not influence the targeting of PEX26 into the ER membrane. But PEX19 is known to stabilize PMPs just after their translation in the cytosol^2^,^45^,^46^. Indeed, we observed that the pool of non-glycosylated PEX26 was stabilized in the absence of Get3. When expressed in a Δ*pex19*Δ*get3* strain the amount of non-glycosylated PEX26 decreased compared to the protein detected in the Δ*get3* strain. Degradation of non-targeted PEX26 is likely proteasome dependent as the addition of a proteasome inhibitor increased the amount of detectable PEX26. These results allow drawing a model whereby Pex19 is able to stabilize cytosolic PEX26 in the absence of Get3 (Fig. 6). If Pex19 exerts an additional influence on PEX26 targeting, this is likely to occur after ER integration or in a parallel pathway.

We showed that yeast Pex15 and mammalian PEX26 behave similar despite being expressed in evolutionarily distant organisms. In yeast, mammalian PEX26 appears to follow a similar itinerary like Pex15. We speculate that PEX26 is included in the same pre-peroxisomal vesicles that require Pex19 to bud from the ER and to be targeted to peroxisomes^38^. These vesicles might correspond to the structures closely associated with the ER that we observed while expressing PEX26 in Δ*pex19* cells.

### The role of the ER in peroxisome formation

Most cellular membrane proteins are first targeted to the ER. Chloroplasts and mitochondria, owing to their endosymbiotic history, are noticeable exceptions. Peroxisomes are not of endosymbiotic origin and seem to have evolved a more complex set of protein targeting routes^12^,^51^,^52^. Therefore, whether PMPs are first targeted to the ER, is discussed controversially in the field.

It has often been argued that post-translational import of proteins into peroxisomes and growth and division of peroxisomes speak for direct import of both, matrix and membrane proteins^4^,^52^,^53^. On the other hand, biogenesis of peroxisomes in yeast occurs mainly *de-novo*^54^ at the ER from distinct pre-peroxisomal vesicles that contain partially assembled docking and RING complexes^11^. Upon fusion of these vesicles the transport complex required for peroxisome matrix protein import is established. A prerequisite for peroxisome maturation is that PMPs travel via the ER towards the budding side of these pre-peroxisomal vesicles. While nearly all of PMPs in *S. cerevisiae* enter the ER^47^,^48^, in human cells, only PEX3, PMP34, and PEX16 have been shown to pass the ER^55^,^56^,^57^.

Recent work in a mammalian system provided evidence that PEX3 and PMP34 pass the ER before entering the peroxisome if PEX16 is present^57^. It was proposed that PMPs are transported to the peroxisome either using a PEX16- and ER-dependent (group I) pathway, or a direct, PEX16-indendent pathway with direct post-translational PMP insertion into the peroxisomal membrane (group II). Both pathways are not mutually exclusive – upon saturation of the slower group I pathway PEX3 was directed to the peroxisome via the other^52^,^57^. Following this model, PEX26 could be targeted PEX16-dependently via the ER towards the peroxisome^50^. When PEX26 is overexpressed, the PEX16-dependent pathway might be saturated and the successive employment of the PEX16-independent path could explain why we find both glycosylated and non-glycosylated PEX26 on our Western blots and autoradiographs. We also showed that similar to mammalian PEX3, PEX26 in yeast can travel two possible routes to the peroxisome. The GET-dependent targeting to the ER is favored. But if this pathway is impaired, a second, Pex19-dependent targeting mechanism ensures proper integration into the peroxisomal membrane (Fig. 6).

## Materials and Methods

### DNA cloning

All oligonucleotides and plasmids used in this study are listed in Supplementary Table S2 and Supplementary Table S3, respectively. pENTR221-PEX26^co^ (PST1193) was derived from pENTR221-PEX26 (PST1147) by using the primers OST751, OST752, OST753, OST754, OST755, OST756, OST757, OST758, OST789, OST790, OST791, and OST792 for site-directed mutagenesis of pENTR 221-PEX26.

pAG416-GPD-EGFP-PEX26^co^ (PST1310) was generated from entry vector pENTR 221-PEX26^co^ (PST1193) and destination vector pAG416-GPD-EGFP-ccdB (PST1117) by using the Gateway system. pAG416-GPD-EGFP-PEX26 (PST1311) was obtained similarly by combining pENTR 221-PEX26 (PST1147) with pAG416-GPD-EGFP-ccdB (PST1117). pAG416-GAL1-EGFP-PEX26^co^ (PST1486) was obtained by recombination of pENTR221-PEX26^co^ (PST1193) and pAG416-GAL1-EGFP-ccdB (PST1341).

pRS416GAL1-PEX26^co^-EcoRI-opsin (pJB3) was cloned in two steps. First olignonucleotides coding the opsin-tag (OST921 and OST922) were annealed and cloned into the vector pRS416GAL1 opened by EcoRI and XhoI, yielding pRS416GAL1-opsin (pJB2). PEX26 was amplified from PST1311 using oligonucleotides OST957 and OST958 and cloned into XbaI and EcoRI sites of pJB2. For cloning of pcDNA3.1(-)-PEX26-EcoRI-opsin (PST1362) the XbaI-XhoI fragment containing PEX26-EcoRI-opsin from pJB3 was introduced into pcDNA3.1(-) using the same restriction sites.

For cloning of pRS416GAL1-PEX26^co^-opsin PEX26^co^ was amplified from PST1310 with the oligonucleotides OST957 and OST1088 and inserted into XbaI and XhoI of pRS416GAL1 (PST904). pRS416GAL1-EGFP(pJB26) and pRS416TEF-EGFP (pJB31) were derived from pRS416GAL1 and pRS416TEF (PST814) respectively by insertion of EGFP, amplified from PST1310 using primers OST562 and 1272 into XbaI and EcoRI sites. In order to obtain pRS416GAL1-EGFP-PEX26^co^-opsin (pJB27) and pRS416TEF-EGFP-PEX26 252–305, PEX26^co^ and PEX26 252–305 were amplified from pJB8 using OST1273/OST1274 and OST1301/OST1303. The resulting PCR products were inserted into EcoRI and XhoI sites of pJB26 and pJB36.

To obtain p416TEF-PEX15ΔTMD-PEX26ΔSTOP (PST1413) the N-terminal domain of Pex15 and the TMD and C-terminus of PEX26 were amplified from PST1127 and PST1310 using OST1101 and 1103 and OST1102 and 958, respectively. These PCR products then served as PCR template for the amplification of the Pex15-PEX26 fusion protein using primers OST1101 and 958. The product was cloned into the XbaI and EcoRI sites of p416TEF (PST814). Sequencing showed a frame shift in the Pex15-PEX26 fusion region. The frame shift was corrected by site-directed mutagenesis using the oligonucleotides OST1275 and 1276. OST1176 and 1177 were used for adding a STOP codon by site-directed mutagenesis. The final product was p416TEF-PEX15ΔTMD-PEX26-STOP (PST1565). For cloning of pcDNA3.1(−)-PEX26-opsin (PST1380) PEX26 was amplified from PST1147 using primers OST957 and 1052. The PCR product was cloned into the vector XbaI and XhoI sites of pcDNA3.1(−) (PST994).

PEX15 and PEX15ΔTMD were amplified from genomic DNA using the primers OST746 and 747 and OST746 and 748, respectively. The amplificates were integrated into pDONR221 by the BP-reaction. The resulting constructs were pENTR221-PEX15 (PST1326) and pENTR221-PEX15ΔTMD (PST1566). PST1326 and PST1566 were recombined with pAG416-GPD (PST1340) resulting the expression clones pAG416-GPD-PEX15 (PST1126) and pAG-GPD-PEX15ΔTMD (PST1127). pEXP-Venus-PEX15 (PST1133) and pEXP-Venus-PEX15ΔTMD (PST1134) were obtained by recombining pENTR221-PEX15 (PST1326) or pENTR221-PEX15ΔTMD and pDEST-N-Venus (PST1183) in an LR-reaction.

For cloning pCR3.1-Myc-Pex15 (PST1137) and pCR3.1-PEX15ΔTMD (PST1138) PEX15 and PEX15ΔTMD were amplified with using the primers OST746 and 747 and OST746 and 748. The PCR product was integrated into pCR3.1-Myc by BP- reaction.

For cloning of pRS425-Sec63-RFP (PST811) SEC63-RFP was excised from pSR1960 (PST802) using NotI (blunted) and HindIII restriction enzymes, and inserted into pRS425 (PST809) SacI (blunted) and HindIII sites.

For cloning of pcDNA3.1-TRC40_GRmyc, the coding sequence of TRC40_GR was amplified from pQE80-MBP-TRC40_GR using the primers Asna1-F and Asna1myc-R. The PCR product was cloned into the pcDNA3.1/V5-His-TOPO vector according to the manufacturer’s instructions (Invitrogen).

### Immunofluorescence and microscopy

Cells expressing *PEX26* or *PEX15* constructs were analyzed by direct fluorescence and immunofluorescence experiments. If not indicated otherwise, cells were transfected for 48 hours using Effectene (Qiagen). Fixation, permeabilization, incubation with antibodies, and mounting was carried out as described^58^,^59^. The following antibodies were used: anti-PEX14 (ProteinTech, rabbit polyclonal, dilution 1:200) and anti-Myc (Cell Signaling, mouse monoclonal, dilution 1:2000), anti-Catalase (Oxis International, rabbit polyclonal, 1:1000), anti-mouse Alexa488 (MoBiTech, dilution 1:200), and anti-rabbit Cy3 (Jackson ImmunoResearch, dilution 1:200). Yeast cells were mounted in low-melting agarose for imaging. Samples were analyzed using a 100x oil immersion objective (NA 1.3) on a Zeiss Imager M1 fluorescence wide field microscope with a HRm Camera and the Axiovision 4.8 acquisition software. For yeast microscopy widefield images were contrast-enhanced and false-colored in blue to show cell contours.

### [^35^S] pulse label experiments

HeLa cells were transfected as indicated. 24 hours after transfection, cells were washed once with PBS. Cells were incubated with 2 ml RPMI cell medium (Sigma-Aldrich), 10% freshly dialyzed fresh bovine serum and 1% glutamine for 2 hours at 37 °C. Cells were labelled for 15 min or 30 min at 37 °C using 50 μCi/well [^35^S]-methionine and [^35^S]-cysteine (Hartmann Analytic, Braunschweig) and then washed twice with PBS. For lysis, cells were treated with RIPA buffer (20 mM Tris-HCl, pH 7.4, 150 mM sodium chloride, 2 mM EDTA, 1% NP40, 1 mM dithiothreitol (DTT), 0.1 mM PMSF, 1 x Complete protease inhibitors (Roche)) for 10 min on ice. Cell debris was removed by centrifugation (10 min at 20,000 g and 4 °C). The supernatant of lysate was supplemented with 20 μl of anti-opsin antibody^60^ cell culture supernatant (courtesy of Stephen High) and was incubated for 1 hour at 4 °C on a turning wheel. Incubation continued for another 3 hours after addition of 20 μl ProteinG-agarose (Thermo Scientific). Samples were centrifuged at 5,200 × g at 4 °C for 3 min. The supernatant was discarded and beads were washed five times with RIPA buffer and once with PBS buffer containing 150 mM sodium chloride. Beads were eluted in SDS gel loading buffer (40 mM Tris-HCl pH 6.8, 2% SDS, 0.12 M DTT, 5% glycerol, 0.025% bromophenol blue). The proteins were separated by SDS-PAGE on a polyacrylamide gel. Gels were vacuum dried on Whatman paper. Phosphoimager screens were exposed for ca. 2–25 hours and analyzed by a Fujifilm base 1800 II camera. Intensities of protein bands were quantified using ImageJ (NIH).

### Yeast stains and yeast cell culture

Yeast strains used in this study are listed in Supplementary Table S4. To obtain Δ*pex19*Δ*get3* the *PEX19* ORF was deleted in Δ*get3* by integration of the *hphNT1* marker using OST526, OST527 and pFA6-hphNT1 as described^61^.

The following yeast media were used in this study: Synthetic drop out medium (0.67% yeast nitrogen base without amino acids (Difco), 0.75% potassium chloride, 0.5% ammonium sulfate, 0.083% amino acids, carbon source as indicated), oleate medium (0.15% oleate, 0.9% amino acids (all amino acids), 0.1% yeast extract, 0.17% yeast nitrogen base, 0.4% Tween40 and 2% agar). The amino acid mixture contained 5.2% adenine hemisulfate, 5.2% arginine-HCl, 5.2% histidine-HCl, 15.7% homoserine, 5.2% isoleucine, 5.2% leucine, 5.2% lysine-HCl, 5.2% methionine, 7.9% phenylalanine, 7.9% tryptophan, 5.2% tyrosine, 3.1% uracil and 23.6% valine. Amino acids used as selection marker were left out in the respective amino acid mixture. Solid media contained 20 g/l agar.

For galactose induced expression yeasts were first cultured in a liquid synthetic medium containing 2% glucose. Cells were then diluted to an OD_600_ of 0.1 and grown in synthetic medium containing 4% raffinose overnight to an OD_600_ of 4. Cells were transferred to synthetic medium containing 2% galactose for induction of expression. In pulse-chase-experiments, protein expression was induced for 2.5 hours, and chase was initiated by shifting cells to medium containing 2% glucose.

For the oleate growth assays cells were cultured in a synthetic medium containing 2% glucose for 10 hours. Yeast cultures were diluted to an OD_600_ of 0.1, transferred to synthetic medium containing 0.3% glucose, and incubated for ten hours and grown to an OD_600_ of three. Samples were diluted to an OD_600_ of 0.1 and starved on 0.3% glucose medium overnight under same conditions. Yeasts were spun down, washed three times with sterile water, and a 10-fold dilution series ranging from an OD_600_ of 1 to 10^−3^ was prepared. Yeasts were then spotted onto oleate plates and incubated for one week at 28 °C.

### Cell lysis and Western blot analysis

Yeast cells equivalent to 3 OD_600_ were harvested 15 min, 30 min, 120 min and 150 min after induction. Cells were washed with TE buffer (10 mM Tris-HCl, pH 8.0, 1 mM EDTA), resuspended in 500 μl water and immediately lysed by addition of 75 μl lysis concentrate (5 M sodium hydroxide, 1% beta-mercaptoethanol, 100 mM PMSF, 0.5 M EDTA). Proteins were precipitated by 23% trichloroacetic acid for 5 min one ice. After centrifugation at 13.000 x g for 15 min at 4 °C the pellets were washed twice with 100% acetone (−20 °C). Pellets were resuspended in 20 μl water, and 10 μl loading buffer (4x Roti-Load1 buffer (Roth)) was added. Samples were denaturated for 5 min at 95 °C.

Mammalian cells were washed twice with PBS. 70 μl 4x SDS-loading buffer (including 1× complete protease inhibitors (Roche) and 9 mM N-ethylmaleinimid (NEM) without beta-mercaptoethanol) were added to the cells. After 10 min incubation at room temperature 20 mM DTT was added. Cells were removed from the cell culture plates and lysed by ultrasound three times for 60 s (Bandelin SonoRex RK 106 S, 480 W). Samples were incubated for another 30 min at 37 °C and for 10 min at 95 °C. For the experiment described in Fig. 2a cells were washed twice with PBS containing 1 mM EDTA. 24 hours after transfection, cells were removed from culture plates and centrifuged at 20,000 g for 10 min at 4 °C. Pellets were resuspended and lysed in RIPA lysis buffer. Cells were incubated for 30 min one ice and were vortexed several times during this incubation step. Then the samples were centrifuged at maximum speed for 15 min at 4 °C. Supernatants were used for Western blot. 4x SDS-loading buffer was added. Control samples were treated with PNGase (New England Biolabs) or with EndoH (New England Biolabs) for deglycosylation.

Proteins were separated by SDS-PAGE on a 12% polyacrylamide gel and transferred to a nitrocellulose membrane by semi-dry blotting. The following antibodies were used: mouse anti-opsin^60^ (courtesy of Stephen High), mouse anti-PGK (Life technologies), mouse anti-GFP (BD Biosciences), chicken anti-Pex19 (Jackson ImmunoResearch) mouse anti-alpha-tubulin (Sigma Aldrich), donkey anti-mouse IgG HRP-conjugated (Jackson ImmunoResearch). All primary antibodies were diluted 1:1000, except anti-PGK 1:10000). Secondary antibodies were used at 1:10000 dilution. Chemiluminescence was generated with Lumi-light and Lumi-light plus Western blotting substrates (Roche) and recorded using the Luminescent image analyzer LAS 4000 (Fuji). Intensities of protein bands were quantified using ImageJ (NIH).

## Additional Information

**How to cite this article**: Buentzel, J. *et al.* Conserved targeting information in mammalian and fungal peroxisomal tail-anchored proteins. *Sci. Rep.* **5**, 17420; doi: 10.1038/srep17420 (2015).

## Supplementary Material

Supplementary Information
