# Supplementary material for: Conserved targeting information in mammalian and fungal peroxisomal tail-anchored proteins
Source: Sci Rep. 2015 Dec 2;5:17420. doi: 10.1038/srep17420 (PMC4667187; doi:10.1038/srep17420)
Supplement: Supplementary Information [file srep17420-s1.pdf]

Supplementary Information

Conserved targeting information in mammalian and fungal peroxisomal tail-anchored proteins

Judith Büntzel, Fabio Vilardi, Amelie Lotz-Havla, Jutta Gärtner, Sven Thoms

Supplementary Tables

Table S1. Statistical analysis of glycosylation.
Table S2. Oligonucleotides used in this study
Table S3. Plasmids used in this study
Table S4. Yeast strains used in this study

Supplementary Figures

Figure S1. Full-size uncropped Western blots and autoradiographs of all figures.
Figure S2. Gray-scale representations of single channel images of figures 1h, i and 3a, b.
Figure S3. Targeting of PEX26 TMD and C-segment to the peroxisome in yeast.

Supplementary Tables

	Fig.	Gel lane	Mean (%)	S.e.m.	P value
Wild-type	4a	2	69	3.9	
DAmP-Sec62	4a	3	73	4.4	0.5100
Δsec72	4a	4	74	3.5	0.4700
Δget2	4a	5	15	2.7	<0.0001
Δget3	4a	8	13	1.6	<0.0001
Δget5	4a	6	18	3.4	<0.0001
Δget1/2	4a	7	17	5.2	<0.0001
Δget1/2/3	4a	9	20	7.8	0.0002
Δpex19	5a	2	91	3.2	0.0018
Δpex19 Δget3	5c	5	44	1.5	0.0039

Table S1. Quantification of glycosylation of PEX26 expressed in yeast strains

**Name**	**Sequence** (5' to 3')
OST526	AAGTATTGACGGAAAGAAGAAATGCCAAACATACAACACGAAGTACGTACGCTGCAGGTCGAC
OST527	TTACTGTTATCATAAATATATATACCTTATTGTTGTTTGCAACCGATCGATGAATTCGAGCTCG
OST562	GGCCGAATTCCTTGTACAGCTCGTCCATG
OST751	GTCCCGGCCCGCGCGCCGGCCGTG
OST752	CACGGCCGGCGCGCGGGCCGGGAC
OST753	CCTGGACTTCCGCGCGGCGCTGGAG
OST754	CTCCAGCGCCGCGCGGAAGTCCAGG
OST755	GACCTGCGAGCGCGCCTGGCAGAGTC
OST756	GACTCTGCCAGGCGCGCTCGCAGGTC
OST757	GGCAGAAATGGATCGCTGGCAAGAAGTCCTC
OST758	GAGGACTTCTTGCCAGCGATCCATTTCTGCC
OST789	GAATTTCACGTGCAGAGGGTGCTGCTGCCTC
OST790	GTACATCCAGTCGCCTCTCCTCACCAAAGG
OST791	CTCTTCCGCTGGATCAGGAAGGCTGCATTTTC
OST792	GAAAATGCAGCCTTCCTGATCCAGCGGAAGAG
OST921	AATTCGGTCCAAACTTCTACGTCCCATTCTCCAACAAGACCGGTTAG
OST922	TCGACTAACCGGTCTTGTTGGAGAATGGGACGTAGAAGTTTGGACCG
OST957	ACTAGTTCTAGAATGAAGAGCGATTCTTCGACC
OST958	CGCGCGAATTCGTCACGGATGCGGAGCTG
OST1052	GCGCCTCGAGCTAGCCCGTCTTGTTGGAGAA
OST1088	CGCGCTCGAGCTAACCGGTCTTGTTGGAGAATGGGACGTAGAAGTTTGGACCCGGACGGATGCGGAG
OST1101	GCGCTCTAGAAGTGCTGCAAGTGAGATAAT
OST1102	GCATCATTTCACAAGAAGTCAAAAAGAGTCTCCTGGCTG
OST1103	GCCAGGAGACTCTTTTTGACTTCTTGTGAAATGATGC
OST1176	CAGCTCCGCATCCGTGACTGAGAATTCGGTCCAAACTTC
OST1177	GAAGTTTGGACCGAATTCTCAGTCACGGATGCGGAGCTG
OST1194	GGAAGGCTGCATTTTCTGCCCTCTACCAGCTCCGAATCCG
OST1195	CGGATGCGGAGCTGGTAGAGGGCAGAAAATGCAGCCTTCC
OST1196	CTCGCCTCTACCAGCTCGCCATCGCTGACGGTCCTAACTTCTACG
OST1197	CGTAGAAGTTAGGACCGTCAGCGATGGCGAGCTGGTAGAGGCGAG
OST1272	CGCGTCTAGAATGGTGAGCAAGGGCGAG
OST1273	CGCGGAATTCATGAAGAGCGATTCTTCGAC
OST1274	CGCGCTCGAGCTAACCGGTCTTGTTGGA
OST1275	GAAGCATCATTTCACAAGAAGTAAAAAGAGTCTCCTGGCTGCC
OST1276	GGCAGCCAGGAGACTCTTTTTACTTCTTGTGAAATGATGCTTC
OST1301	GCGCGAATTCTTCAAAAAGAGTCTCCTGGCT
OST1303	CGCGCTCGAGCTAGTCACGGATGCGGAG
Asna1-F	GAATTCTCCACCATGGCGGCAGGGGTG
Asna1myc-R	CTACTACAAGTCCTCTTCAGAAATGAGCTTTTGCTCCTGGGCACTGGGGGGCTT

Table S2. Oligonucleotides used in this study

Number	Name	Source
	pFA6-hphNT1	Janke et al.[61]
PST802	pSM1960	Metzger et al.[64]
PST809	pRS425	Mumberg et al.[65]
PST811	pRS425-SEC63-RFP	This study
PST814	p416TEF	Mumberg et al.[65]
PST904	pRS416GAL1	Mumberg et al.[65]
PST994	pcDNA3.1(-)	Invitrogen
	HcRed-SKL	E. Hettema[4]
PST1117	pAG416-GPD-EGFP-ccdB	Alberti et al.[66]
PST1126	pAG416-GPD-PEX15	This study
PST1127	pAG416-GPD-PEX15ΔTMD	This study
PST1133	pEXPVenusPEX15	This study
PST1134	pEXPVenus-PEX15ΔTMD	This study
PST1135	pCR3.1-Myc-PEX26	This study
PST1137	pCR3.1Myc-PEX15	This study
PST1138	pCR3.1Myc-PEX15ΔTMD	This study
PST1147	pENTR221-PEX26	This study
PST1165	pDONR207	Invitrogen
PST1173	pcDNA3.1-TRC40_WTmyc	Horst et al.[38]
PST1174	pcDNA3.1-TRC40_SW1myc	Horst et al.[38]
PST1183	pDEST-N-Venus	Alberti et al.[66]
PST1193	pENTR221-PEX26^{co}	This study
PST1310	pAG416-GPD-EGFP-PEX26^{co}	This study
PST1311	pAG416-GPD-1EGFP-PEX26	This study
PST1326	pENTR221-PEX15	This study
PST1340	pAG416-GPD-ccdB	Alberti et al.[66]
PST1341	pAG416-GAL1-EGFP-ccdB	Alberti et al.[66]
PST1362	pcDNA3.1(-)-PEX26-EcoRI- opsin	This study
PST1380	pcDNA3.1 (-)-PEX26-opsin	This study
PST1413	p416TEF-PEX15ΔTMD-PEX26ΔSTOP	This study
PST1414	pcDNA 3.1-TRC40_GR	This study
PST1486	pAG416-GAL1-EGFP-PEX26^{co}	This study
PST1565	pRS416TEF-PEX15ΔTMD-PEX26	This study
PST1566	pENTR221 PEX15ΔTMD	This study
	pQE80-MBP-TRC40_GR	Favaloro et al.[67]
	pcDNA3.1/V5-His-TOPO	Invitrogen
	pDONR221	Invitrogen
	pCR3.1-Myc	Invitrogen
pJB2	pRS416GAL1-EcoRI-opsin	This study
pJB3	pRS416GAL1-PEX26^{co}-EcoRI-opsin	This study
pJB8	pRS416GAL1-PEX26^{co}opsin	This study

pJB26	pRS416GAL1-EGFP	This study
pJB27	pRS416GAL1-EGFP-PEX26^{co}-opsin	This study
pJB31	p416TEF-EGFP	This study
pJB36	p416TEF-EGFP-PEX26 253-305	This study

Table S3. Plasmids used in this study

**Strain**	**Source**
Wild-type BY4741	Euroscarf (Frankfurt)
Δget1/ Δget2/ Δget3	Schuldiner et al.[22]
Δget1/ Δget2	Schuldiner et al.[22]
Δget3	Schuldiner et al.[22]
Δget5	Yeast Consortium Deletion Library[68]
Δpex15	Yeast Consortium Deletion Library[68]
Δpex19	Yeast Consortium Deletion Library[68]
Δsec72	Yeast Consortium Deletion Library[68]
DAmP-*sec62*	Schuldiner et al.[63]
Δpex19Δget3	This study

Table S4. Yeast strains used in this study

Fig. 1 (b)

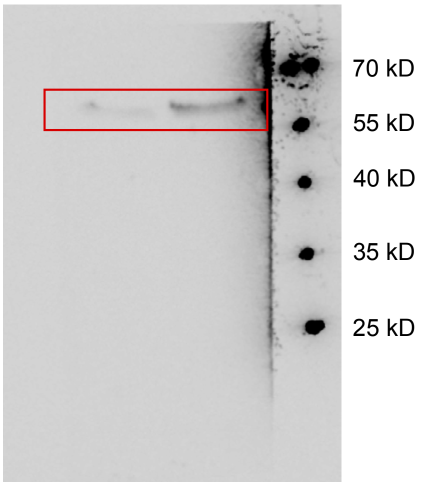

anti-GFP

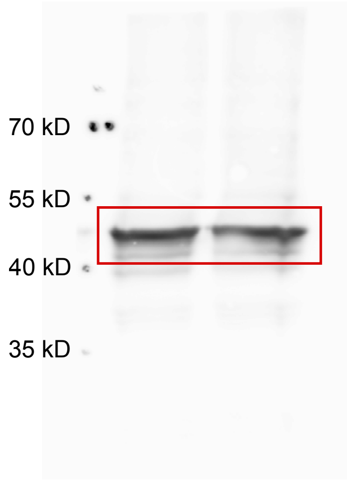

anti-PGK

Fig. 2 (a)

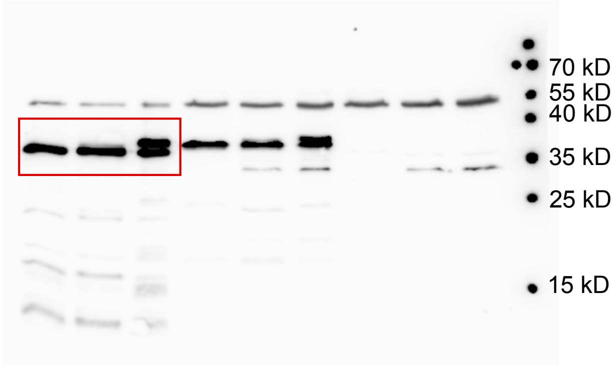

PEX26-opsin

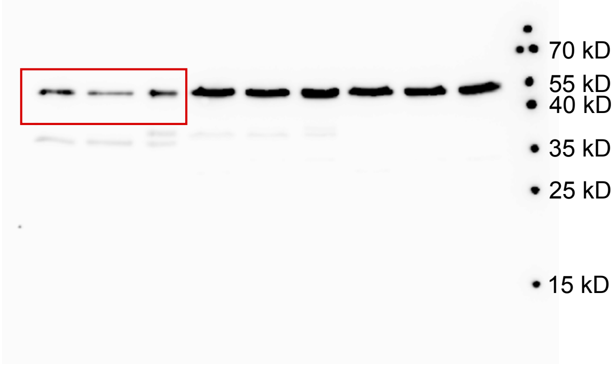

alpha-tubulin

Fig. 2 (b)

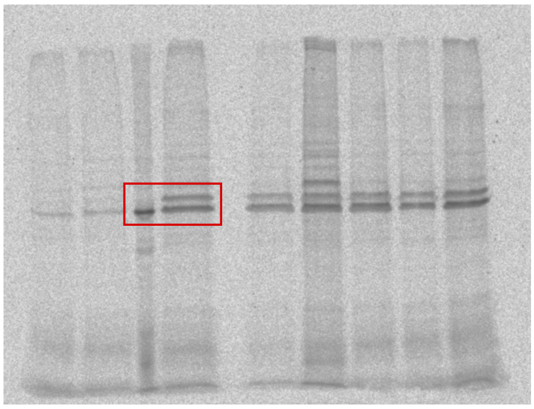

autoradiograph

Fig. 2 (c)

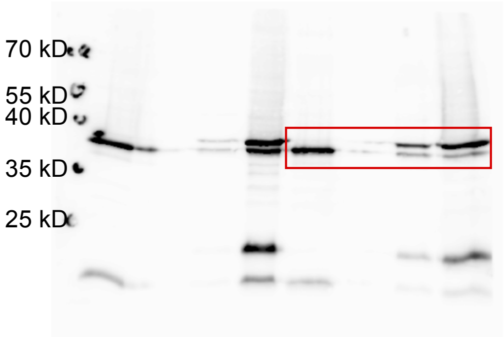

anti-opsin

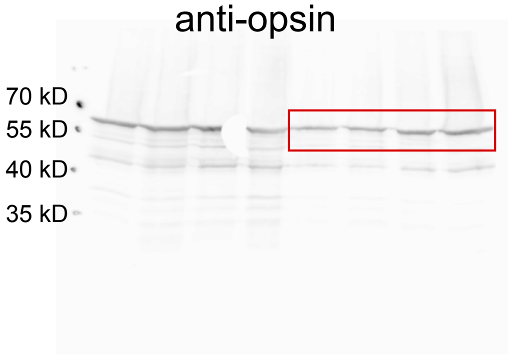

anti-PGK

Fig. 4 (a)

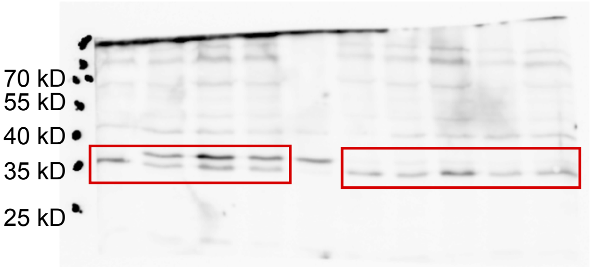

anti-opsin

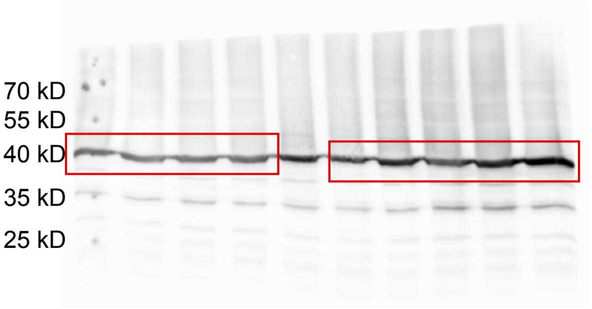

anti-PGK

Fig. 4 (d)

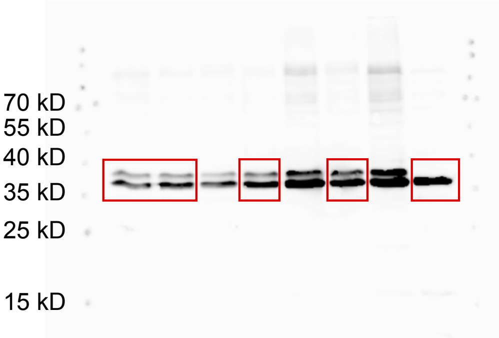

anti-opsin

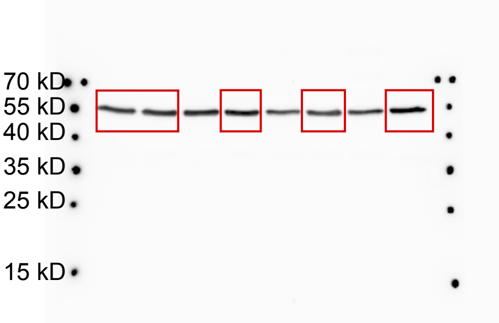

anti-PGK

Fig. 4 (b)

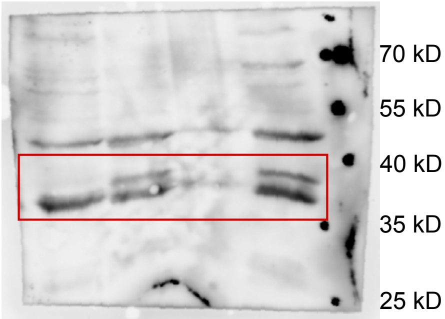

anti-opsin

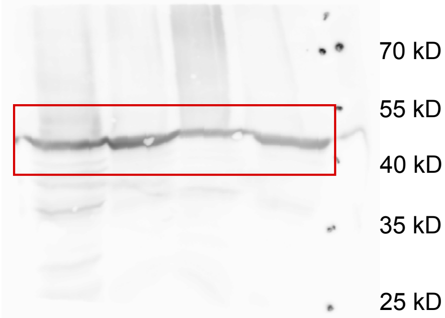

anti-PGK

Fig. 4 (e)

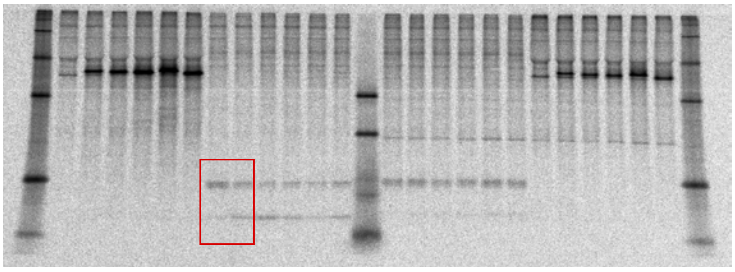

autoradiograph

Fig. 4 (f)

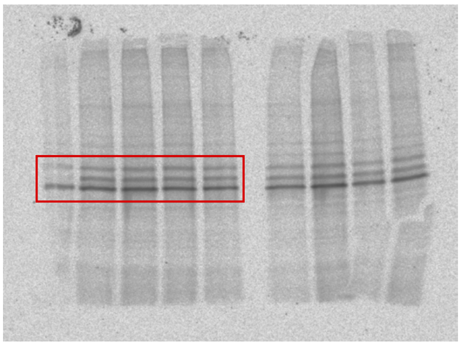

autoradiograph

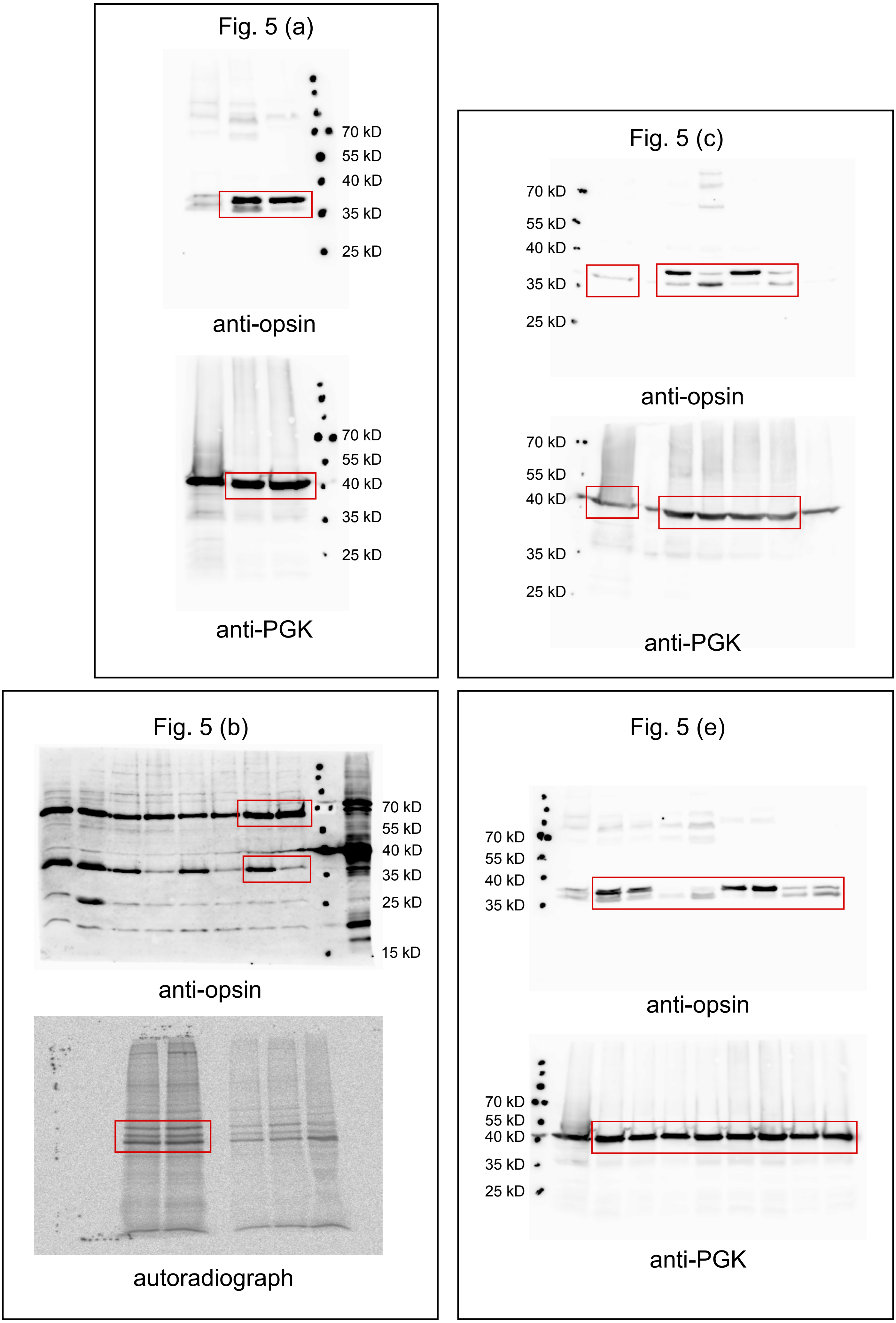

Figure S1. Full-size uncropped Western blots and autoradiographs of all figures.

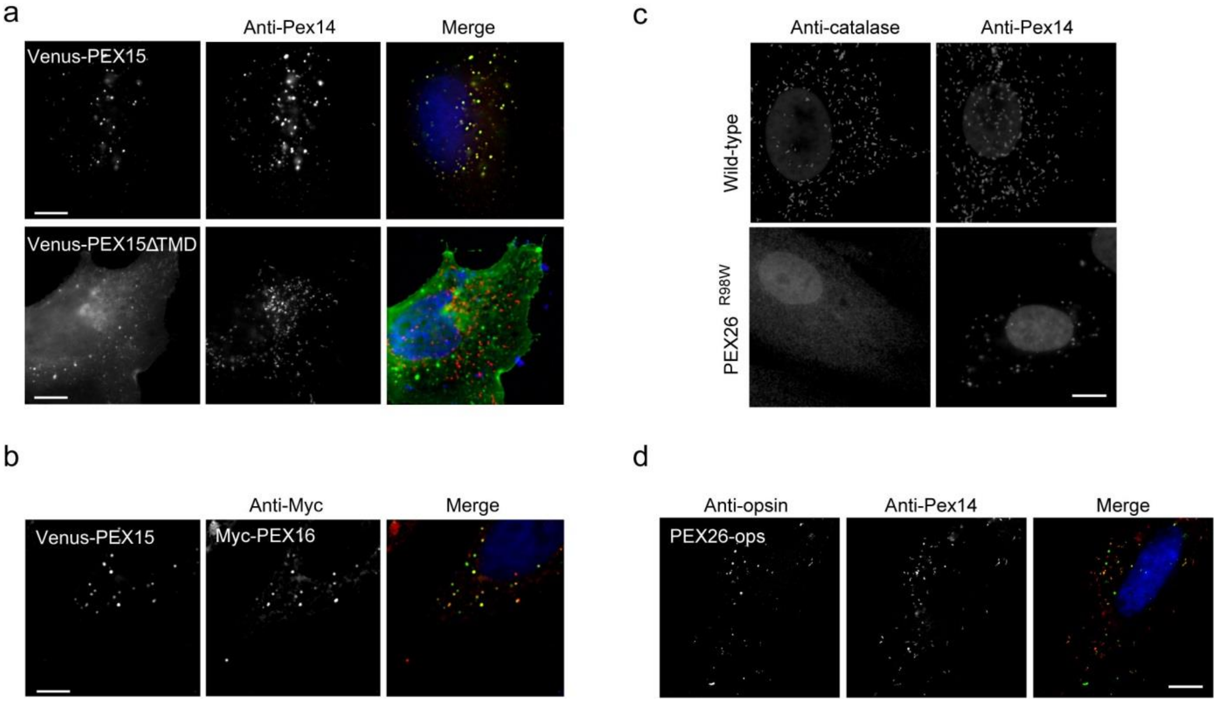

Figure S2. Gray-scale representations of single channel images of figures 1h, i and 3a, b.

(a) Yeast Pex15 localizes to peroxisomes in HaLa cells. Peroxisome targeting is dependent on TMD and luminal segment of Pex15. Direct fluorescence of Venus-Pex15 and Venus-Pex15ΔTMD shows co-localization with the peroxisome marker PEX14 (immunofluorescence) and cytosolic localization, respectively. (b) PEX26 and Pex15 are recruited to the same population of peroxisomes. Direct fluorescence of Venus-Pex15, immunofluorescence of Myc-PEX26, both proteins co-localize. Bar = 10 µm. (c) Deficiency in PEX26 leads to impaired peroxisome maturation. Fibroblasts of a control and a patient carrying a $PEX26^{R98W}$ mutation were stained for the peroxisomal matrix protein catalase and the PMP PEX14. Catalase import is impaired in patient fibroblasts, peroxisomes are less abundant and larger than in wild-type. Bar = 10 µm. (d) Expression of Myc-PEX26 in $PEX26^{R98W}$ fibroblasts partially rescues peroxisome maturation and import of catalase. Bar = 10 µm.

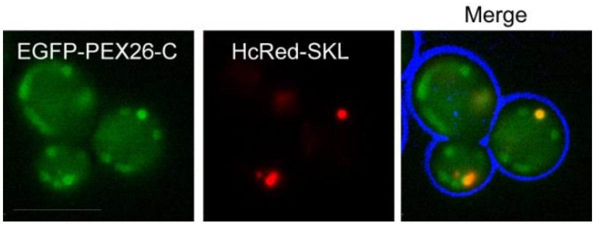

Figure S3. Targeting of PEX26 TMD and C-segment to the peroxisome in yeast.

(a) Conserved peroxisomal targeting information of PEX26 resides in the TMD and the luminal C-segment. PEX26-C, a truncated version of PEX26 (amino acids 252 to 305), was fused to N-terminal EGFP. Constitutive, TEF promoter-driven expression in wild-type yeast. EGFP-PEX26-C puncta partially co-localize with the peroxisomal marker HcRed-SKL.
